# Decreased insight, but not self-stigma or belief about medicine, is associated with greater severity of delusions in a sample of long-stay patients with schizophrenia: a cross-sectional study

**DOI:** 10.1186/s12888-023-04711-1

**Published:** 2023-04-03

**Authors:** Christina Beainy, Chadia Haddad, Feten Fekih-Romdhane, Souheil Hallit, Georges Haddad

**Affiliations:** 1 Faculty of Sciences, Lebanese University, Fanar, Lebanon; 2 Research and Psychiatry Departments, Psychiatric Hospital of the Cross, P.O. Box 60096, Jall-Eddib, Lebanon; 3 INSPECT-LB (Institut National de Santé Publique, d’Épidémiologie Clinique et de Toxicologie- Liban), Beirut, Lebanon; 4 School of Health Sciences, Modern University for Business and Science, Beirut, Lebanon; 5 School of Medicine, Lebanese American University, Byblos, Lebanon; 6 The Tunisian Center of Early Intervention in Psychosis, Department of Psychiatry “Ibn Omrane”, Razi Hospital, Manouba, 2010 Tunisia; 7 Faculty of Medicine of Tunis, Tunis El Manar University, Tunis, Tunisia; 8 School of Medicine and Medical Sciences, Holy Spirit University of Kaslik, P.O. Box 446, Jounieh, Lebanon; 9 Applied Science Research Center, Applied Science Private University, Amman, Jordan

**Keywords:** Insight, Delusions, Auditory hallucinations, Self-stigma, Attitudes towards medication, Schizophrenia

## Abstract

**Background:**

There are, to date, limited and inconsistent findings concerning the relationship between insight and psychotic symptoms, despite some evidence in favor of the clinical and therapeutic relevance of the insight construct. We aimed to add to the pool of the available data in this area, by examining the correlations between the severity of insight and positive psychotic symptoms (delusions and auditory hallucinations), while accounting for self-stigma and attitudes towards medication, in a sample of long-stay inpatients with schizophrenia.

**Methods:**

A cross-sectional study was conducted at the Psychiatric Hospital of the Cross, between July and October 2021. A total of 82 patients diagnosed with schizophrenia (aged 55.55 ± 10.21 years, 54.9% males) were enrolled. The semi-structured psychotic symptom rating scales, the Birchwood Insight Scale, the Belief About Medicine Questionnaire, and the Internalized Stigma of Mental Illness were used.

**Results:**

The mean duration of illness in years was 30.15 ± 11.73, and the mean duration of hospitalization in years was 17.56 ± 9.24. Sixteen out of the 82 patients (19.5%) were considered as having poor insight. Bivariate analyses showed that higher chlorpromazine equivalent dose was significantly associated with more delusions, whereas higher insight was significantly associated with lower delusions. Multivariable analyses revealed that Higher chlorpromazine equivalent dose (Beta = 0.004) was significantly associated with more delusions, whereas higher insight (Beta = − 0.89) was significantly associated with less delusions. No significant associations were found between insight, self-stigma and hallucinations.

**Conclusion:**

Our results imply that more impaired insight is associated with greater severity of delusions, above and beyond the effects of self-stigma and medication doses. These findings are valuable to aid clinicians and researchers improve their understanding of the relationship insight-psychotic symptoms, and could help personalize prevention and early intervention strategies in schizophrenia.

## Introduction

Over the past three decades, there has been a growing research interest in the concept of insight in psychotic disorders, mainly due to its high clinical relevance [1]. Insight has been shown to be at least partly lacking in a large proportion of patients with schizophrenia [2, 3]. According to Davis [4], insight refers to the awareness of having a mental illness, the ability to attribute psychotic symptoms (delusions and hallucinations) to a mental illness, and recognition of the need for medication. All these three abilities appear to reflect executive functioning, thereby insinuating that impaired insight represents impairment of executive function [5]. In this line, studies using functional imagery have shown that each insight dimension is mediated by specific brain regions [6]. Another theoretical perspective regarded insight as a primary feature or symptom of the disease. This conceptualization has later been criticized for being too narrow, given the multidimensionality and complexity of the insight construct, and its multiple related factors (including emotional state, cognition/meta-cognition, illness stage, sociocultural processes) [5]. These different explaining models might explain the reason for limited and inconclusive evidence on the relationship between insight and clinical outcomes. Indeed, insight appear to be linked to multiple adverse clinical outcomes [7]. Several meta-analyses have documented that poor insight is related to impaired quality of life [8], cognitive function [9, 10], social cognitive abilities [10, 11], as well as to several symptom domains [10, 12]. Insight has been found to negatively influence adherence to antipsychotics [13, 14], uptake of psychotherapy [15], and long-term illness outcome [13]. In addition, there is some evidence that good insight contributes to a better prognosis over and above treatment adherence; however, this remains hard to establish [13].

So far, the vast majority of prior research focused on neurocognitive or social functioning in relation to insight in psychoses, while little is known about how psychotic symptoms relate to insight [16]. However, the relationship between insight and psychopathology symptoms is rather complex, with only scant studies having investigated the interplay between these variables [17], and having led to controversial findings [18]. While some studies have found a significant negative association between insight and positive symptoms (e.g., [10]), others could demonstrated no [19] or only a small relationship [18]. A meta-analysis by Mintz et al. [18] concluded that there is only a small negative association between insight and psychotic symptoms, and that acute patient status moderated this association. In other words, the negative correlation between insight levels and positive symptoms severity seems to be stronger in acutely psychotic patients [18]. In contrast, Quee et al. [16] found that insight was correlated with clinical symptoms over and above neurocognitive functioning in patients with multiple episode psychosis, but not in those with recent-onset psychosis. A longitudinal study showed that insight is improved when acute symptoms are reduced or resolved [20]. These conflictual findings add support to the fact that the relationship between insight and psychotic symptoms is multi-determined and still unclear, which emphasizes the need for additional studies to further elucidate its nature.

Insight is not the only factor associated with positive symptoms. Therefore, another confounding factor that could also be taken into consideration when assessing this relationship is known as ‘self-stigma’, which refers to a combination of three related problems: a lack of knowledge (ignorance and misinformation); negative attitudes (prejudice); and excluding or avoiding behaviors (discrimination) [21]. In fact, negative stereotypes and discrimination of mental disorders are still very prevalent worldwide [22], including in Arab countries [23]. Self-stigma causes patients to reject their diagnosis and refuse treatment, even when they are conscious of their symptoms [24]. The “why try” effect suggests that self-stigma may interact with mediators such as self-esteem and self-efficacy, to engender reluctance to participation in rehabilitation services; because patients perceive themselves as “not worthy” of achieving any personal goals or aspirations [25]. Having good insight into schizophrenia was shown to be linked to increased self-stigma [26], and self-stigma was found to be associated with an increased severity of positive symptoms [27, 28]. Besides self-stigma, another important factor that might affect both insight and psychotic symptoms is patients’ attitudes towards medication. Previous research has documented significant associations between medication attitudes and psychotic symptoms [29, 30]. In addition, holding favorable attitudes about medication has consistently been shown to correlate with a better insight [13, 29, 31–33]. More particularly, Wiesjahn et al. [33] found that more positive attitudes towards medication were significantly associated with greater endorsement of biological causal attributions, higher attribution of the symptoms to a mental illness, and increased sense of treatment need. Haq et al. [30] found that attitudes to medication negatively correlated with delusions, and positively correlated with insight in patients diagnosed with schizophrenia and schizoaffective disorder. Therefore, attitudes to medication is considered a potential moderating factor when it comes to good insight.

The impact of insight on the severity of delusions and auditory hallucinations is still undergoing research studies. To our knowledge, no previous studies have investigated insight levels with regards to positive psychotic symptoms among patients with schizophrenia in Lebanon, a culture that is specifically influenced by strong stigmatization towards the mentally ill for lack of mental health literacy [34]. The objective of this study was to examine the correlations between the severity of insight and positive psychotic symptoms (delusions and auditory hallucinations), while accounting for self-stigma and attitudes towards medication, in a sample of long-stay inpatients with schizophrenia. By including only long-stay patients, we reduce the bias due to a potential confounder (medication compliance), given that all inpatients adhere to medication-taking recommendations made by their healthcare providers. We hypothesized that insight levels will be significantly associated with delusions and hallucinations severely, over and above self-stigma and medication attitudes.

## Methods

### Study design and participants

**Fig. 1:**
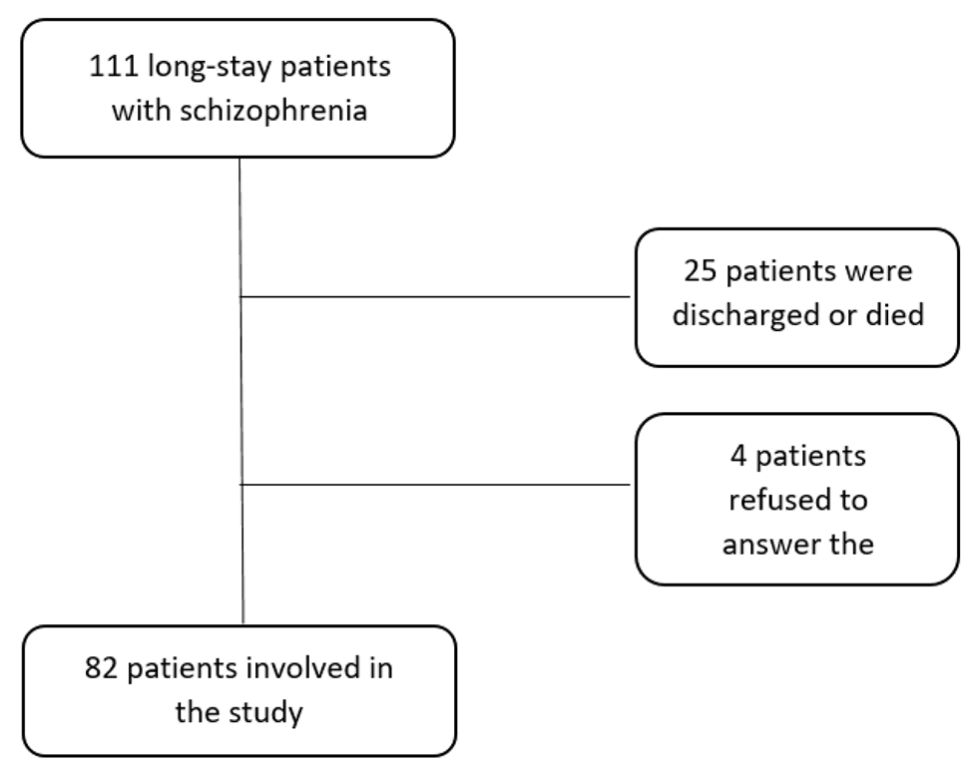
A flow chart of the enrolled participants

A cross-sectional study was conducted between July and October 2021. Our target population were patients with schizophrenia who were in a long-stay accommodation in the Psychiatric Hospital of the Cross, Jal Eddib (suburbs of the capital Beirut), Lebanon. To be eligible, chronic inpatients should: (1) be aged over 18 years, (2) be diagnosed with schizophrenia by two independent psychiatrists according to the DSM-5 criteria [35], (3) have received the schizophrenia diagnosis at a young age, and have been in the above-mentioned long-stay hospital for more than two years, and (4) be able to recognize the objectives of the study and give their free consent to participate (in case of inability to consent a family member did). A total of 82 patients were enrolled in the study (Fig. 1).

### Minimum sample size

We used G*Power software to determine the minimum sample size; the latter was 64 participants, considering an alpha error of 5%, a power of 80%, and moderate effect size f^2^ of 0.3 and allowing 10 predictors to be included in the final models.

### Measures

Data collection was conducted in face-to-face interviews. All data was gathered by the same investigator, who was trained in conducting the evaluations using the semi-structured psychotic symptom rating scales (PSYRATS). The investigator was an independent researcher, and had no previous contact with participants. Data related to socio-demographic factors (age, gender, marital status, education, family income, family history of psychiatric illness) as well as duration of illness, length of hospitalization, and chlorpromazine equivalent dose were collected from the hospital database. The following four measures have been administered to all participants:

#### The PSYRATS

This is a semi-structured scale that quantifies the severity of auditory hallucinations and delusions [36]. It is divided into two subscales with each item being rated from 0 (absent) to 4 (severe): The auditory hallucinations subscale (AHS) has eleven items: frequency, duration, controllability, loudness, location; severity and intensity of distress; amount and degree of negative content; beliefs about the origin of voices; and disruption. For this scale, a 4-dimensional model is used with these variables: Distress (negative content, distress, and control), Frequency (frequency, duration, and disruption), Attribution (location and origin of voices), and Loudness (loudness item only). The delusions subscale (DS) has six items: duration and frequency of preoccupation; intensity of distress; amount of distressing content; conviction and disruption. This subscale is a 2-dimensional model assessing these variables: Distress (amount/intensity) and Frequency (preoccupation, conviction, and disruption). Total scores can range from 0 to 24, with higher scores representing more severe delusions. Total scores on these factors can range from 0 to 8 and 0–16 respectively, with higher scores representing more severe delusions. In this study, the Cronbach alpha value was 0.938.

#### The birchwood insight scale (BIS)

The BIS is an 8-items scale that measures the three main dimensions of insight (ability to re-label symptoms, awareness of mental illness, and recognition of a need for treatment) [37]. It is a 3-point response format (agree - unsure – disagree). The eight items of BIS are organized into three subscales; ‘Awareness of illness’ (2 items), ‘Re-labeling of symptoms’ (2 items), and ‘Need for treatment’ (4 items). Each subscale has a mean score from 0 to 4. The subscale scores can be summarized to a total score (range 0–12), where a higher score indicates more insight. Scores ≥ 9 imply good insight. The BIS has shown to be valid and reliable for assessing insight in patients with schizophrenia [38]. In this study, the Cronbach alpha value was 0.648.

#### The belief about medicine questionnaire (BMQ)

The BMQ [39] consists of two five-item scales that oppose two variables: ‘Necessity’ and ‘concerns’. ‘Necessity’ is the belief about the importance of prescribed medication for controlling their disease. ‘Concerns’ reflects a person’s worry about potential adverse consequences of taking the prescribed medication. Degree of agreement is rated from 1 = strongly disagree to 5 = strongly agree. Total scores for the Necessity and Concerns Scales range from 5 to 25 with higher scores indicating stronger beliefs. In this way, the necessity (benefits) and cost (concern) are weighed against each other.

The original BMQ contains another set of questions that fall into the ‘general belief’ that is opposed to the ‘specific’ one used in this study. General belief relates to a person’s thoughts about general medications and prescriptions, and wasn’t used for the reason that the patients don’t possess the mental capacity of answering these ‘theoretical’ questions as the BMQ is not specific for the psychiatric/mental units. We used the Arabic validated version of the BMQ, which demonstrated good reliability and validity [40]. In this study, the Cronbach alpha value was 0.658.

#### The internalized stigma of mental illness (ISMI)

The ISMI (27) is a 29-item questionnaire measuring self-stigma among persons in the clinical psychiatric population. It is composed of five subscales (Alienation, Stereotype Endorsement, Discrimination Experience, Social Withdrawal, and Stigma Resistance). Each item is rated from 1 (strongly disagree) to 4 (strongly agree). For the interpretation of scores, we used the two-category method where: 2.51–4.00 reports high internalized stigma, and any score below it does not indicate a significant internalized stigma. The Arabic ISMI used in this study showed adequate psychometric properties [41]. In this study, the Cronbach alpha value was 0.923.

### Statistical analysis

IBM SPSS Statistics for Windows, version 25.0 (IBM Corp., Armonk, N.Y., USA) was used to perform the data analysis. A descriptive analysis was performed, where categorical variables were expressed as absolute frequencies and percentages and quantitative variables as means and standard deviations. The hallucinations, delusions, insight and Belief About Medicine Questionnaire scores were considered normally distributed since their skewness and kurtosis values varied between − 1.96 and + 1.96 [42]. The ISMI score was not normally distributed, with its skewness and kurtosis falling outside this range. The log transformation did not show a normal distribution as well. Consequently, the score was dichotomized according to the median. The independent-sample t-test was used to compare continuous variables between groups, whereas the ANOVA was used to compare three or more means. Pearson correlation test was used to evaluate the association between continuous variables.

Bonferroni correction was applied to take into account multiple comparisons; the corrected p value was 0.006, which was calculated by dividing 0.05 by the total number of variables in the analysis (= 9). Two linear regressions were carried out taking auditory hallucinations and delusions scores as dependent variables respectively.

Models were adjusted over variables that showed significance (after Bonferroni correction) in the bivariate analysis. A p < 0.05 was considered significant in the final models.

## Results

### Sample description

Table 1 shows the demographic and other characteristics of patients. The mean age of the patients was 55.55 ± 10.21 years, with 54.9% males. The majority (89.0%) were single, with low education level (complementary level and below: 79.3%), and low-income level (80.2%). Only 29.3% have a family history of psychiatric illness. Mean duration of illness in years was 30.15 ± 11.73, and the mean duration of hospitalization in years was 17.56 ± 9.24. The mean chlorpromazine equivalent dose was 957.69 ± 947.36. Sixteen out of the 82 patients (19.5%) were considered as having poor insight according to a cut-off score of 9 or above on the BIS.

**Table 1:** Sociodemographic and other characteristics of the participants (N = 82)

Variable	N (%)
Gender	
Male	45 (54.9%)
Female	37 (45.1%)
**Marital status**	
Single/divorced/widowed	73 (89.0%)
Married	9 (11.0%)
**Education level**	
Illiterate	15 (18.3%)
Primary (≤ 6 years of school)	15 (18.3%)
Complementary (7–10 years of school)	35 (42.7%)
Secondary (11–13 years of school)	14 (17.1%)
University	3 (3.7%)
**Socioeconomic level**	
Low (Less than 1000 USD)	68 (80.2%)
Intermediate (1000–2000 USD)	15 (18.5%)
High ( More than 2000 USD)	1 (1.2%)
**Family history of psychiatric illness**	
Yes	24 (29.3%)
No	58 (70.7%)
	**Mean ± SD**
**Age (in years)**	55.55 ± 10.21
**Duration of illness (in years)**	30.15 ± 11.73
**Duration of hospitalization (in years)**	17.56 ± 9.24
**Chlorpromazine equivalent dose (mg)**	957.69 ± 947.36

### Description of the scales used

The scales used in the current study are described in Table 2.

**Table 2:** Description of the median, mean, SD, and the range of the scales used in this study

	Median	Mean	SD	Min	Max	Skewness	Kurtosis
The Psychotic Symptom Rating Scales (PSYRATS)							
PSYRATS -The auditory hallucinations subscale	21.50	19.02	13.16	0	42	− 0.232	-1.072
PSYRATS -The delusions subscale	8.00	9.12	8.53	0	33	0.610	− 0.549
**Birchwood Insight Scale**	9.00	8.43	3.88	0	16	0.080	− 0.728
**Belief About Medicine Questionnaire**							
Necessity scale	19.00	17.48	6.76	5.00	25	− 0.595	− 0.885
Concerns scale	10.50	11.92	5.55	5.00	24	0.648	− 0.686
**The Internalized Stigma of Mental Illness**	62.00	64.93	9.29	52	102	1.832	4.030

### Bivariate analysis

Tables 3 and 4 show the bivariate analysis taking auditory hallucinations and delusions scores as the dependent variables. Higher chlorpromazine equivalent dose was significantly associated with more delusions, whereas higher insight was significantly associated with lower delusions.

**Table 3:** Bivariate analysis taking the hallucination and delusion scores as dependent variables

	Auditory hallucinations	Delusions
	Mean ± SD	Mean ± SD
Gender		
Male	20.20 ± 14.68	9.33 ± 9.00
Female	17.59 ± 11.05	8.86 ± 8.03
*p*	0.363	0.806
**Marital status**		
Single/divorced/widowed	20.21 ± 13.14	9.57 ± 8.64
Married	9.33 ± 8.91	5.44 ± 6.94
*p*	0.018	0.172
**Education level**		
Illiterate	22.20 ± 14.33	9.93 ± 8.76
Primary	18.20 ± 12.50	10.40 ± 9.03
Complementary	20.08 ± 12.48	9.65 ± 8.84
Secondary	16.07 ± 14.05	7.50 ± 7.29
University	8.66 ± 15.01	0.01 ± 0.01
*p*	0.453	0.342
**Family history of psychiatric illness**		
Yes	16.83 ± 12.88	8.08 ± 8.36
No	19.93 ± 13.27	9.55 ± 8.63
*p*	0.335	0.482
**Internalized stigma towards mental illness**		
Low	16.15 ± 13.13	11.36 ± 8.54
High	20.96 ± 12.96	7.61 ± 8.28
*p*	0.105	0.050

**Table 4:** Correlation matrix of continuous variables

	1	2	3	4	5	6	7	8	9
1. Hallucinations	1								
2. Delusions	0.24	1							
3. Belief About Medicine (necessity)	0.28	− 0.19	1						
4. Belief About Medicine (concern)	0.09	− 0.08	− 0.05	1					
5. Age	− 0.20	0.07	− 0.23	0.02	1				
6. Duration of illness	− 0.21	0.09	− 0.19	0.13	**0.71**	1			
7. Duration of hospitalization	0.11	0.11	− 0.10	0.16	**0.47**	**0.49**	1		
8. Chlorpromazine equivalent dose	0.11	**0.44**	0.03	0.04	− 0.05	0.12	− 0.05	1	
9. Insight	0.28	**− 0.34**	**0.47**	0.01	**− 0.34**	− 0.26	0.04	− 0.03	1

Values in bold indicate significant *p* values after Bonferroni correction.

### Multivariable analysis

None of the variables was significantly associated with auditory hallucinations scores (Table 5, Model 1).

**Table 5:** Multivariable analyses

	Unstandardized Beta	Standardized Beta	*p*	95% CI
Model 1: Linear regression (ENTER method) taking the auditory hallucinations score as the dependent variable (Nagelkerke R^2^ = 0.114)				
Age	− 0.01	− 0.004	0.980	− 0.41; 0.40
Duration of illness	− 0.17	− 0.15	0.334	− 0.51; 0.18
Insight	0.98	0.21	0.074	− 0.10; 2.05
Self-stigma (high vs. low*)	3.25	0.12	0.276	-2.64; 9.14
**Model 2: Linear regression (ENTER method) taking the delusions score as the dependent variable (Nagelkerke R**^**2**^ **= 0.315)**				
Marital status (married vs. single*)	-2.20	− 0.08	0.419	-7.57; 3.18
Chlorpromazine equivalent dose	0.004	0.40	**< 0.001**	0.002; 0.005
Insight	− 0.89	− 0.30	**0.003**	-1.47; − 0.31
Self-stigma (high vs. low*)	-1.87	− 0.11	0.287	-5.34; 1.60

*Reference group; numbers in bold indicate significant *p* values.

Higher chlorpromazine equivalent dose (Beta = 0.004) was significantly associated with more delusions, whereas higher insight (Beta = − 0.89) was significantly associated with less delusions (Table 5, Model 2).

## Discussion

This study was motivated by the limited and inconsistent findings concerning the relationship between insight and psychotic symptoms, despite some evidence in favor of the clinical and therapeutic relevance of the insight construct. We aimed to add to the pool of the available data in this area, by investigating the association between insight levels and delusions/hallucinations severity, while taking into account other possible confounding factors. Our results imply that more impaired insight is associated with greater severity of delusions, above and beyond the effects of self-stigma and medication doses. No significant associations were found between insight, self-stigma and hallucinations. These findings are valuable to aid clinicians and researchers improve their understanding of the relationship insight-psychotic symptoms, and could help personalize prevention and early intervention strategies in schizophrenia.

Our sample displayed a relatively low prevalence of poor insight (19.5%), which may be explained by the fact that all patients are in long stay in the hospital, and regularly receive psychoeducation sessions [43]. additionally, chronic and multiple episode patients have been found to generally exhibit more awareness of their distress, and a better ability to attribute it to a mental illness compared to first episode patients [16]. The main finding of the present study was that poorer insight remained significantly associated with delusions, but not with hallucinations, after accounting for main confounders in the multivariable analyses. Consistent with our findings, some previous studies have found a significant relationship between lack of insight and higher severity of delusions [16, 44]. A previous study showed that delusions were associated with low self-reflectiveness and high self-certainty, reflecting low cognitive insight, whereas hallucinations were associated with high self-reflectiveness and low self-certainty, reflecting high cognitive insight [16]. We may think that, as patients gain understanding on the pathological aspect of their false ideations, they are abler to discard some thoughts as non-valid, and question themselves about the ‘delusional’ aspect of their beliefs. The more severe the patient’s delusional state is, the less interested they are in seeking help for lack of insight. The psychoanalytic literature has formalized the concept of defense mechanisms, which ward off painful emotions stemming from awareness. Primitive defenses, such as denial or projection, are particularly implicated in reality distortion and delusions [45]. As such, lack of insight (i.e., denial of the illness) would protect the individual from the painful awareness of being mentally ill. Parellada et al. [20] longitudinally surveyed a sample of adolescent recent-onset psychosis patients, and found that insight negatively correlated with symptom severity, and improved during follow-up in parallel with symptom improvement; leading authors to suggest insight as a “trait” which becomes apparent once symptoms are no longer prominent. Following this line of reasoning, the association between insight and psychopathology symptoms seem to be more pronounced outside of the acute phases [16], which further supports our current findings.

With regard to the lack of significance in the relationship between insight and hallucinations, our findings are contrary to some research that was done in schizophrenia patients. For instance, a study suggested that self-certainty about hallucinations, meaning less skepticism around the voices, makes it harder for patients to realize the pathological aspect of their disease, creating therefore a lowered insight [46]. In other terms, a patient who is certain that their hallucinations are coming from an external source – more severe – will not be able to judge themselves as having a disease, and this state by itself, is the definition of a lacking insight. Another previous study found that patients with chronic, persistent, more severe auditory hallucinations had more impaired insight compared to those with no or episodic hallucinations [47]. Authors suggested that, it is not the hallucination itself but rather its persistence which is related to insight; and that “getting used” to the hallucination could lead to ‘‘believing’’ in its real existence [47]. Other research has shown that insight is differently associated with external (heard outside the head) vs. internal (heard inside) auditory hallucinations [48]; albeit, here again, mixed results have been reported [49]. A review by Waters et al. [50] suggested that the presence of insight appears to influence individuals’ views, interpretation and response to auditory hallucinations; and therefore contributes to determining the meaning of these experiences. In sum, insight into schizophrenia seems to interact with delusions and hallucinations in a complex way. Additional research distinguishing different types and contents of delusions and hallucinations in patients with various clinical characteristics may confirm (or infirm) our findings and these previous assumptions.

### Limitations

The present study represents a first attempt to correlate the variables previously discussed, but results of our study should be considered keeping in account certain limitations. It was designed as a cross sectional study in the clinical setting. The stability as well as causality of the relationships need to be re-examined using a longitudinal study design and a larger sample size. We only included chronic patients who were in a long-stay in the hospital, which limits the generalizability of the findings to outpatients, and those with early-onset and acute phases of the illness. In terms of future research, it would be useful to extend the current findings by investigating novel metacognitive therapies that can also be studied for the progression of illness severity over time with the amelioration of insight and diminishment of self-stigmatization of patients suffering from psychosis.

### Clinical implications

Antipsychotic medication is today the cornerstone of treatment of delusions, combined with psychotherapy for a better outcome. For severe cases that appear to be drug-resistant, Clozapine can be a drug of choice, and if it did not give its expected outcome, electro convulsive therapy and transcranial magnetic stimulation can be used [51]. Despite these treatment options, many patients with delusions show non-responsiveness to antipsychotics [52, 53]. Targeting other potential contributing factors to delusions, such as insight, sheds light on possible alternative approaches to treat medication-refractory delusions in schizophrenia patients. In this regard, new approaches have been suggested for the treatment of psychosis targeting insight (e.g., Metacognitive Reflection Insight Therapy [54], metacognitive training [55]). Interventions that tackle metacognitive abilities by means of a narrative, meaning-oriented approach have shown promising results in terms of improving awareness in patients with severe psychotic disorders [56–59]. Besides, other neurobiological approaches are also targeting psychosis by the use of possible therapeutic interventions that target stimulation of relevant brain areas, aimed at promoting insight in psychotic patients [60]. However, more experimental research is still needed before any conclusions can be drawn.

## Conclusion

Despite pharmacotherapy of schizophrenia-spectrum disorders has known important advances during the past decades, a large proportion of patients show treatment-resistance. Even if preliminary and to be confirmed in future longitudinal studies involving a larger number of patients, the current findings add support to previous suggestions that insight is inversely associated with delusions severity, above and beyond the effects of self-stigma. Therefore, given the importance of insight in clinical outcomes, we call for future longitudinal and experimental research to determine directionality in the associations between insight, delusions, hallucinations, attitudes toward medication and self-stigma; and to test whether metacognitive-based psychotherapies are effective in improving insight and positive psychotic symptoms in schizophrenia.

## Data Availability

The datasets generated during and/or analyzed during the current study are available from the corresponding author on reasonable request.

## Abbreviations

BIS: Birchwood Insight Scale
BMQ: Belief About Medicine Questionnaire
ISMI: Internalized Stigma of Mental Illness
PSYRATS: The semi-structured psychotic symptom rating scales
